# Interkingdom multi-omics analysis reveals the effects of nitrogen application on growth and rhizosphere microbial community of Tartary buckwheat

**DOI:** 10.3389/fmicb.2023.1240029

**Published:** 2023-09-14

**Authors:** Qingcheng Qiu, Dabing Xiang, Qiang Li, Hanlin Wang, Yan Wan, Qi Wu, Xueling Ye, Liangzhen Jiang, Yu Fan, Bingliang Liu, Yanxia Liu, Han Li, Changying Liu

**Affiliations:** ^1^Key Laboratory of Coarse Cereal Processing, Ministry of Agriculture and Rural Affairs, Sichuan Engineering and Technology Research Center of Coarse Cereal Industralization, Chengdu University, Chengdu, China; ^2^School of Food and Biological Engineering, Chengdu University, Chengdu, China; ^3^Guizhou Academy of Tobacco Science, Guiyang, China

**Keywords:** Tartary buckwheat, nitrogen, rhizosphere bacteria, rhizosphere fungi, interkingdom multi-omics

## Abstract

Tartary buckwheat (*Fagopyrum tataricum* Gaertn.) is an important pseudocereal crop with excellent edible, nutritional and medicinal values. However, the yield of Tartary buckwheat (TB) is very low due to old-fashioned cultivation techniques, particularly unreasonable application of nitrogen fertilizer. To improve the understanding on the theories of nitrogen use in TB, the effects of nitrogen application on growth, as well as chemical properties and microbial community of rhizosphere soil were investigated in this study. Nitrogen application could promote the plant height, stem diameter, nitrogen accumulation and yield of TB. The relative abundance and diversity of bacteria and fungi in the rhizosphere soil of TB were improved by nitrogen fertilizer. Nitrogen application increased the abundance of beneficial bacteria such as *Lysobacter* and *Sphingomonas* in rhizosphere soil, and decreased the abundance of pathogenic fungi such as *Fusarium* and *Plectosphaerella*. The results indicated that nitrogen application changed the distribution of microbial communities in TB rhizosphere soil. Furthermore, the specific enriched or depleted microorganisms in the rhizosphere soil of four TB varieties were analyzed at OTU level. 87 specific nitrogen-responsive genes with sequence variation were identified in four varieties by integrating genomic re-sequencing and transcriptome analysis, and these genes may involve in the recruitment of specific rhizosphere microorganisms in different TB varieties. This study provided new insights into the effects of nitrogen application on TB growth and rhizosphere microbial community, and improved the understanding on the mechanisms of TB root–microbe interactions.

## 1. Introduction

Tartary buckwheat (*Fagopyrum tataricum* Gaertn.) is a dicotyledonous plant belonging to the *Polygonaceae* family and *Fagopyrum* genus, which is one of the main cultivated varieties of buckwheat (*Fagopyrum* spp.) (Luthar et al., 2020, 2021; Ye et al., 2022a). Tartary buckwheat (TB) has been used as the raw material to produce various products since it has the properties of both food and medicine (Zhu, 2016; Zou et al., 2021; Ye et al., 2022b). TB is abundant in protein, lipids, flavonoids, polyphenols, rutin, vitamins and resveratrol, which are beneficial for human health (Yan et al., 2022; Liu et al., 2023b). However, the yield of TB is very low, typically below 1,500 kg/hm^2^, which was difficult to meet the market demand (Jiang et al., 2022). The low yield of TB is mainly due to old-fashioned cultivation techniques in TB producing areas, particularly unreasonable nitrogen application. Therefore, reasonably applying nitrogen fertilizer and improving nitrogen use efficiency are the important approaches to improve TB yield. Additionally, the knowledge on the mechanism of nitrogen absorption and utilization of TB should be improved.

Nitrogen was one of the important constituent elements of macro molecular substances such as proteins and nucleic acids, and was also one of the large elements required for plant growth and development (Franche et al., 2009). Therefore, sufficient nitrogen supply during plant growth was crucial for plant growth and development. Increasing nitrogen fertilization has always been one of the effective agricultural measures to increase crop yield. Previous studies have confirmed that rational nitrogen fertilization can significantly increase the yield of rice (*Oryza sativa* L.), wheat (*Triticum aestivum* L.), sorghum (*Sorghum bicolor* L.) and other crops (Li et al., 2022c; Liao et al., 2022). However, unreasonable nitrogen application will not only reduce the yield and quality of crops, but also cause serious adverse problems, including increasing of production costs, higher soil acidification, reduced quality of cultivated land and environment pollution (Liu C. Y. et al., 2021; Yang Y. et al., 2023). A better understanding on the rational nitrogen fertilizer amount and theories of nitrogen use may contribute to improve crop yield.

The rhizosphere was an area composed of plant roots and the surrounding soil, and the microorganisms that interact with plant roots in the rhizosphere were called rhizosphere microorganisms (Chamkhi et al., 2022; Wang et al., 2023). Plant roots can absorb and utilize various nutrients in the soil through rhizosphere microorganisms, and rhizosphere microorganisms can also improve the soil environment and play an important role in plant stress and disease resistance (Liu et al., 2020; Castellano-Hinojosa et al., 2021; Zhou et al., 2023). Numerous studies showed that nitrogen fertilization can change the physical and chemical properties of rhizosphere soil and affect the distribution of microbial communities (Kavamura et al., 2018; Sun et al., 2023b). Nitrogen fertilization has significant effects on fungal communities in soil, rhizosphere and endophytic environment (Bai et al., 2022). Applying nitrogen in the nitrogen-deficient rock soil can increase the relative abundance of nitrogen-fixing bacteria and urease-producing bacteria, thereby improving the soil quality, reducing plant pathogens and increasing the yield of sweetpotato (*Ipomoea batatas* L.) (Ding et al., 2020). In addition, a previous study has also shown that choosing the appropriate ratio of nitrate and ammonium can increase the diversity and strengthen the functions of bacteria in the rhizosphere soil of sesame (*Sesamum indicum* L.) (Wang R. et al., 2022). Thus, discovering the relationship between plants and rhizosphere microorganisms under nitrogen fertilization may provide new approaches to promote the growth and yield of crops.

The microbial community will change the living environment of plants, and the plants will in turn affect the structure of microbial community. The interaction between plants and rhizosphere microorganisms is one of the important factors to maintain ecological balance and develop sustainable ecological agriculture. Therefore, the study of plant-microbe interactions has received extensive attention (Ge et al., 2023; Sun et al., 2023a; Yang H.-J. et al., 2023). Some studies have found that root microbiota is influenced by host genotype. Zhang J. et al. (2019) found that *indica* and *japonica* rice recruit distinct root microbiota, which is depending on the variation of a rice nitrate transporter gene (*NRT1.1B*) by integrating plant metagenomic sequencing and 16S ribosomal RNA gene analysis. Another study showed that rice histone methylation plays a key role in regulating the assembly of root microbiota (Lv et al., 2021). Yu et al. (2021) found that flavonoids mainly promote the enrichment of *Oxalobacteraceae* bacteria in the rhizosphere by measuring the transcriptome of root system and the microbial community of rhizosphere soil, thereby promoting the growth and nitrogen acquisition of maize (*Zea mays* L.). In addition, association studies of GWAS and metagenomics can identify key drivers that affect the assembly of plant-associated microbiota, and individual microbial taxa and genes can also be linked to plant physiology and traits related to plant fitness (Trivedi et al., 2020). It is also necessary to further use interkingdom multi-omics for analyzing the mechanism of crop-microbe interaction.

The effect of nitrogen application on TB is mainly focusing on yield and quality in previous studies, but there were few reports on the research of TB plants–soil/microbe interactions under nitrogen application (Zhang W. L. et al., 2019; Zhang et al., 2021; Gao et al., 2023). Therefore, the effects of different nitrogen application on TB growth, as well as chemical properties, microbial diversity and community distribution of TB rhizosphere soil were investigated in this study. The main purpose of this study was to reveal the diversity and community distribution of TB rhizosphere bacteria and fungi under different nitrogen status. Finally, the TB root–microbial interactions were investigated by interkingdom multi-omics analysis, including genomic re-sequencing, transcriptome and 16S rDNA/ITS high-throughput sequencing. The results of this study can provide guidance for rational nitrogen fertilization during TB planting, and promote the development and yield of TB based on the mechanisms of TB plants–soil/microbe interactions.

## 2. Materials and methods

### 2.1. Experimental site and experimental materials

This study was carried out from March to June 2022 in an experimental field at Chengdu city in Sichuan province, China (104°56′ N, 30°32′ E, 387 m a.s.l.). The climate of this station is mid-subtropical humid. The mean annual temperature is 17.5°C, and precipitation is 774.1 mm. Four TB varieties, *Fagopyrum tataricum* cv. Yunqiao No. 1 (YQ1), *Fagopyrum tataricum* cv. Yunqiao No. 2 (YQ2), *Fagopyrum tataricum* cv. Xiqiao No. 2 (XQ2) and *Fagopyrum tataricum* cv. Fenghuang (FH), were used in this study. The nitrogen fertilizer (urea contained ≥ 46.0% nitrogen) was purchased from Sichuan Meifeng Chemical Co., Ltd.

### 2.2. Experiment design

The experiments were conducted using a split plot design, and a total of 48 subplots were given. The main plots were assigned to four nitrogen fertilizer concentrations: 0 kg/hm^2^ (N0), 45 kg/hm^2^ (N45), 90 kg/hm^2^ (N90), and 135 kg/hm^2^ (N135). The subplots were assigned to four TB varieties. The area of each plot was 5 m^2^ (2.5 m long, 2 m wide, 8 rows, 30 cm row spacing). The sowing density was set as 1,500,000 plants/hm^2^. Each treatment was repeated three times. The application of phosphate fertilizer (P_2_O_5_) and potassium fertilizer (K_2_O) were 120 kg/hm^2^ and 40 kg/hm^2^, respectively, which were applied as base fertilizer at one time. Nitrogen fertilizer was applied according to base fertilizer: topdressing = 1: 1, and topdressing urea was applied at the flowering stage of TB plants. Disease, insect pests and weed infestation were controlled regularly and timely.

### 2.3. Determination of agronomic traits and grain yield

At the mature stage of TB plants, ten plants were randomly selected in each plot. And then, the plant height, stem diameter, effective branch number, main stem node number, seed number and seed weight of TB were measured and recorded. The SPAD value and nitrogen content of TB leaf were measured by using a plant nutrition analyzer (TYS-4N, Zhejiang Topuyunnong Technology Co., Ltd.).

### 2.4. Soil collection and determination of physical and chemical properties

The rhizosphere soil of TB plants that were cultivated in the N0 and N90 conditions was collected. All the roots of TB plants were carefully dug out, the soil on the surface and large soil lumps at the roots were removed. And then, the soil on the root surface was brushed into a sterilized centrifuge tube by using a sterilized brush (Li et al., 2022a). Finally, the obtained rhizosphere soil was divided into two parts: one half was air-dried at room temperature for the determination of soil physical and chemical properties, and another half was used for detecting microbial community.

The measuring methods of all the chemical properties of soil are as described in a previous reported book (Bao, 2000). The glass electrode method was used to measure the pH of soil, the potassium dichromate oxidation-external heating method was used to measure soil organic content, the alkali-hydrolyzable nitrogen of soil was determined by alkali-hydrolyzable diffusion method, the content of available phosphorus was determined by molybdenum-antimony anti-colorimetric method after extraction with sodium bicarbonate solution, available potassium was determined by ammonium acetate extraction-flame photometer, total nitrogen content was determined by Kjeldahl method with sulfuric acid-accelerator digestion, total phosphorus was determined by NaOH alkali melting and molybdenum-antimony resistance spectrophotometry, and total potassium was determined by NaOH alkali melting and flame photometer (Bao, 2000).

### 2.5. Soil DNA extraction

For the pretreated samples (the rhizosphere soil of YQ1, YQ2, XQ2 and FH), nucleic acid was extracted by OMEGA Soil DNA Kit (D5635-02) (Omega Bio-Tek, Norcross, GA, USA) kit. The quality of DNA was detected by 0.8% agarose gel electrophoresis, and the DNA was quantified by Nanodrop (Thermo Scientific, NC2000).

### 2.6. 16S rDNA high-throughput sequencing

Using 20 ng/μL of the DNA extracted from the rhizosphere soil of YQ1, YQ2, XQ2 and FH as PCR template. The bacterial 16s rRNA V3-V4 region specific primers (338F: 5′-ACTCCTCCTACGGGAGCAGCAGCAMUR-3′; 806R: 5′-GGACTACHVGGGTWTCTAAT-3′) were used for PCR amplification (Xie et al., 2023). The reaction system was as follows: Q5 high-fidelity DNA polymerase (0.25 μL), 5 × reaction buffer (5 μL), 5 × high GC buffer (5 μL), dNTP (10 mM, 2 μL), template DNA (2 μL), forward primer (10 μM, 1 μL), reverse primer (10 μM, 1 μL) and ddH_2_O (8.75 μL). The reaction procedure was as follows: pre-denatured 5 min at 98°C, 25 cycles (98°C, 30 s; 53°C, 30 s; 72°C, 45 s), finally, keep 5 min at 72°C.

The TruSeq Nano DNA LT Library Prep Kit of Illumina Company was used to build the database. And then, the quality of library was checked by using Agilent High Sensitivity DNA Kit on the Agilent Bioanalyzer 2100 system. Finally, for the qualified library, NovaSeq 6000 SP Reagent Kit (500 cycles) was used for carrying out 2 × 250 bp double-terminal sequencing on the Illumina NovaSeq machine. The raw data was submitted to NCBI Short Read Archive database (accession number: PRJNA980195).

The DADA2 plug-in was used for quality filtering, denoising, splicing and chimerism removal of the original data of high-throughput sequencing, and then the characteristic sequence ASVs and abundance data table were generated by merging the filtered data according to 100% sequence similarity. Greengenes database was used to taxonomically annotate ASV, and then the number of microbial groups in different samples at different classification levels (boundary, phylum, class, order, family, genus and species) was compared. Alpha diversity index (Chao1, Shannon and Goods coverage) and beta diversity (PCoA) analysis were carried out by using QIIME2 software.

### 2.7. ITS high-throughput sequencing

A total of 20 ng/μL of DNA extracted from YQ1, YQ2, XQ2 and FH rhizosphere soil was also used as template for PCR amplification of fungal ITS sequence. The fungal ITS1 region primers (ITS5-1737-F: 5′-GGAAGTAAAAGTCGTAACAAGG-3′; ITS2-2043-R: 5′-GCTGCGTTCTTCATCGATGC-3′) were selected for amplification and sequencing (Xie et al., 2023). The PCR reaction system was the same as the above 16S rDNA high-throughput sequencing. The reaction procedure: pre-denatured 5 min at 98°C, 28 cycles (98°C, 30 s; 55°C, 45 s; 72°C, 45 s), finally, keep 5 min at 72°C.

Library quality inspection and sequencing, as well as high-throughput sequenced data processing were the same as the above 16S rDNA high-throughput sequencing. The raw data was submitted to NCBI Short Read Archive database (accession number: PRJNA980190).

### 2.8. Re-sequencing analysis of YQ1 and YQ2

The genome re-sequencing of XQ2 and FH has been determined in our previous study (Liu et al., 2023a). In this study, the genomic data of YQ1 and YQ2 was also re-sequenced. The leaves of YQ1 and YQ2 were removed from the 10-day-old seedlings that were grow in a sterile hydroponic environment by using a dissecting scissors. The leaves were immediately frozen in liquid nitrogen and then stored in a −80°C refrigerator for DNA extraction. The methods of DNA extraction, construction of DNA libraries, Illumina sequencing and data processing were similar to those described in our previous study (Liu et al., 2023a). The raw data of genomic sequencing was submitted to NCBI Short Read Archive database (accession number: PRJNA980146). The genome-wide genetic variants, including single nucleotide polymorphisms (SNPs), insertion/deletions (InDels), copy number variations (CNVs) and structure variations (SVs), were examined by comparing the genomes of YQ1 and YQ2 with reference genome sequence of TB,^1^ and the genes with genome sequence variation were finally generated (Liu et al., 2023a).

### 2.9. Integrating genomic and transcriptome analysis

The genes with genome sequence variation of XQ2, FH, YQ1 and YQ2 were compared by venn diagram analysis, and showing the common and specific varied genes. And then, the expression levels of these genes with genome sequence variation under 10 (N1), 100 (N2), 1,000 (N3) and 10,000 (N4) μM KNO_3_ were analyzed based on the nitrogen-responsive transcriptome data of TB that was reported in our previous study (Liu C. Y. et al., 2021). In addition, gene ontology (GO) enrichment of the differentially expressed genes with genomic variation was analyzed by the GOseq R package.

### 2.10. Statistical analysis

The data of agronomic characters, yield factors and physical and chemical properties of rhizosphere soil of TB were processed by Microsoft Excel 2019, the significant differences were analyzed by SPSS 25.0 software with Duncan multiple-range test, and the basic graphics were made by GraphPad prism 9.

## 3. Results

### 3.1. The agronomic traits of TB under different nitrogen conditions

Nitrogen fertilizer was closely related to agronomic traits (Fang et al., 2018), and the effect of different nitrogen fertilizer amounts on the agronomic traits of TB was explored in this study. As showed in Supplementary Figure 1, nitrogen fertilizer had significant effects on the height and stem diameter of TB plants. With the nitrogen fertilizer rate increased from 0 kg/hm^2^ to 90 kg/hm^2^, the height and stem diameter had increased significantly. TB plants showed no significant change of plant height and stem diameter under nitrogen fertilization ranging from 90 kg/hm^2^ to 135 kg/hm^2^ (Supplementary Figures 1A, B). The number of main stem branches and main stem nodes were not altered by nitrogen addition (Supplementary Figures 1C, D). The SPAD of leaf increased as the nitrogen fertilizer rate increased from 0 kg/hm^2^ to 135 kg/hm^2^ (Supplementary Figure 1E). In addition, the application of nitrogen fertilizer significantly increased the nitrogen content of leaves (Supplementary Figure 1F).

### 3.2. The grain yield of TB under different nitrogen conditions

**FIGURE 1:**
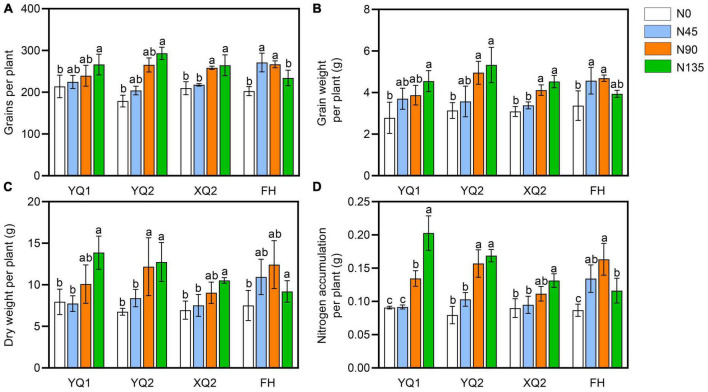
Effect of nitrogen application on grain yield of TB. **(A)** The grains per plant. **(B)** The grain weight per plant. **(C)** The dry weight of TB. **(D)** The nitrogen accumulation per plant. Data are means ± SDs. The means were compared by Duncan’s test. Different treatments marked with different lowercase letters showed significant difference (*P* < 0.05).

It can be seen from Figure 1, the number and weight of grains per plant in XQ2 and FH were significantly increased under the nitrogen fertilizer rate ranging from 0 kg/hm^2^ to 90 kg/hm^2^, while the changes of those was not significant under the nitrogen fertilizer rate ranging from 90 kg/hm^2^ to 135 kg/hm^2^. The dry matter weight and nitrogen accumulation of YQ1, YQ2 and XQ2 significantly increased with the increase of nitrogen application rate (Figure 1). In FH, the nitrogen accumulation decreased under the nitrogen fertilizer rate ranging from 90 kg/hm^2^ to 135 kg/hm^2^ (Figure 1). The above results indicated that nitrogen application has different effects on different varieties of TB, and 90 kg/hm^2^ of nitrogen application could significantly increase the yield per plant of TB. It is suggested that applying 90 kg/hm^2^ of nitrogen fertilizer can improve the agronomic traits and yield of TB.

### 3.3. Physical and chemical properties of rhizosphere soil of TB

**FIGURE 2:**
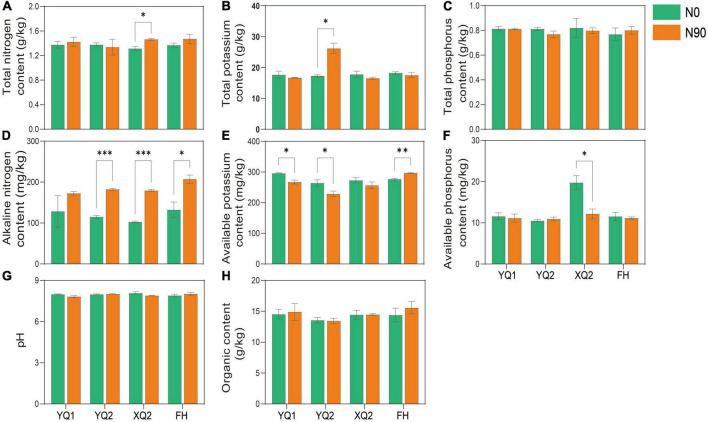
Effects of nitrogen fertilization on nutrient components of TB rhizosphere soil. **(A)** Total nitrogen content. **(B)** Total potassium content. **(C)** Total phosphorus content. **(D)** Alkaline nitrogen content. **(E)** Available potassium content. **(F)** Available phosphorus content. **(G)** pH. **(H)** Organic content. Data are means ± SDs. The means were compared by Duncan’s test. *, **, and *** indicate *P* < 0.05, *P* < 0.01, and *P* < 0.001, respectively.

The soil provides various nutrients for crops, and exogenous nitrogen application may alter the physical and chemical properties of the soil. The properties of TB rhizosphere soil under N0 and N90 were analyzed in this study. As showed in Figure 2, there was no significant difference in the effect of nitrogen fertilizer application on the content of total phosphorus, organic matter and pH in TB rhizosphere soil (*P* > 0.05). Nitrogen application can significantly increase the content of alkali nitrogen in the rhizosphere soil of four TB varieties except YQ1 (Figure 2D). The content of available potassium in YQ1 and YQ2 rhizosphere soil under N90 was significantly lower than that in the soil under N0, while nitrogen application significantly increased the content of available potassium in FH rhizosphere soil (Figure 2E). It is remarkable that N90 significantly increased total nitrogen content (Figure 2A) and reduced available phosphorus content of XQ2 rhizosphere soil (Figure 2F), but their contents showed no significant change in the rhizosphere soil of other three TB varieties. Why XQ2 rhizosphere soil has much higher available phosphorus content under N0 condition? Unfortunately, it cannot be explained based on current data, and more studies are needed to elucidate the interaction between nitrogen and phosphorus in TB rhizosphere soil.

### 3.4. Sequencing data analysis of rhizosphere microorganisms of TB

**FIGURE 3:**
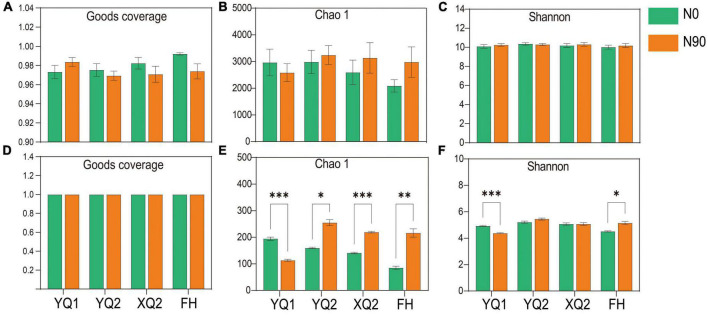
Alpha diversity index of microorganism in different samples. **(A–C)** The Good’s coverage, Chao 1 and Shannon indices of bacteria. **(D–F)** The Good’s coverage, Chao 1 and Shannon indices of fungi. Data are means ± SDs. The means were compared by Duncan’s test. *, **, and *** indicate *P* < 0.05, *P* < 0.01, and *P* < 0.001, respectively.

In this study, high-throughput sequencing technology was used to determine the rhizosphere bacteria of four TB varieties under N0 and N90 conditions. An average of 49,572 raw reads per sample was obtained, and an average of 37,506 clean reads per sample was obtained after removing chimeras and low-quality sequences. Sparse curves were used to determine whether the number of samples sequenced was sufficient, and abundance grade curves to reflect species abundance and uniformity. For bacteria, the number of observed bacterial operational taxonomic units (OTUs) gradually increased with the increase of sequencing reads, and the rarefaction curves flattened when the sequencing reads exceeded 15,000 (Supplementary Figure 2A). It is indicated that the reads sequenced in this study were sufficient to accurately reflect the bacteria structure in the rhizosphere soil of TB. At the same time, the curve begins to smooth when the OTU rank was greater than 1,500, which indicating that the corresponding bacteria in the sample were evenly distributed (Supplementary Figure 2B). In terms of community coverage, the Good’s coverage of all samples was greater than 97%, which indicating that the sequencing results included most of the microorganisms (Figure 3A).

For fungi, an average of 49,453 raw reads per sample was obtained, and an average of 41,101 clean reads per sample was obtained after removing chimeras, low-quality and short sequences. As showed in Supplementary Figure 2C, the number of observed fungal OTUs increased with the increase of sequencing reads. When the sequencing reads exceeds 5,000, all the curves tend to be flattened. When the OTU rank was less than 200, the corresponding curve of some samples decreases rapidly, which indicating that the species distribution was uneven. In addition, the curve began to smooth when OTU rank was greater than 200, which indicating that fungi were evenly distributed in the soil (Supplementary Figure 2D). In terms of sequencing depth index, all samples had high sequencing depth (with an average Good’s coverage index > 0.999) (Figure 3D).

### 3.5. Effects of nitrogen application on alpha diversity index of rhizosphere microorganisms of TB

Chao 1 and Shannon indices were used to calculate the alpha diversity index of rhizosphere soil samples to explain the abundance and diversity of bacterial communities. In terms of abundance indicator, Chao1, nitrogen application increased the bacterial abundance of FH, XQ2, and YQ2 rhizosphere soil, but decreased that of YQ1 (Figure 3B). Besides, the Shannon was slightly improved by nitrogen application (Figure 3C).

In order to study the effect of nitrogen application on rhizosphere fungal communities, the alpha diversity of rhizosphere fungi was measured. Nitrogen application significantly increased the average of Chao1 in the rhizosphere soil of FH, XQ2, and YQ2, but significantly decreased the fungal abundance of YQ1 (*P* < 0.05) (Figure 3E). In terms of community diversity index, nitrogen application significantly increased Shannon in the rhizosphere soil of FH (Figure 3F). Thus, nitrogen fertilizer can increase the fungal diversity in the rhizosphere soils of XQ2 and YQ2, but decreased the diversity of YQ1.

### 3.6. Effects of nitrogen fertilization on rhizosphere microbial community structure of TB

#### 3.6.1. Rhizosphere bacterial community of TB

Based on species annotation and statistical analysis, a total of 25 bacterial phyla, 70 bacterial classes, 215 bacterial families and 323 bacterial genera were detected in all samples, and the variations in abundance of the top 10 bacteria at phylum, class, family and genus levels were listed in the Supplementary Figure 3. *Actinobacteria* (average 36.1%) was the most abundant phylum in all samples, followed by *Proteobacteria* (average 26.8%), *Acidobacteria* (average 14.2%) *Gemmatimonadetes* (average 5.8%), *Chloroflexi* (average 4.7%) (Supplementary Figure 3A). The application of nitrogen fertilizer decreased the abundance of *Actinobacteria* in FH and XQ2 but increased that in YQ1 (*P* < 0.05). Compared with N0, nitrogen application decreased the abundance of *Acidobacteria* in YQ1 and YQ2, and increased the abundance of *Acidobacteria* in FH and XQ2 (*P* < 0.05).

At the genus level, *Subgroup 6* (average 9.3%) was the most abundant genus in all samples (Supplementary Figure 3D), followed by *Nocardioides* (average 3.2%), *Streptomyces* (average 3.1%) and *Amycolatopsis* (average 2.7%). Nitrogen application reduced the relative abundance of *Subgroup 6*, *Nocardioides*, *Streptomyces* and *Amycolatopsis* in rhizosphere samples of YQ1 and YQ2 (*P* < 0.05), the abundance of *Amycolatopsis* and *Nocardioides* in FH and XQ2 samples was also reduced (*P* < 0.05). However, applying nitrogen fertilizer increased the relative abundance of *Subgroup 6* in FH and XQ2 samples (*P* < 0.05).

#### 3.6.2. Rhizosphere fungi community of TB

The variations in abundance of the top 10 fungi at phylum, class, family and genus levels were analyzed (Supplementary Figure 4). At the phylum level, we have identified seven fungi phyla, *Ascomycota* (average 93.4%) was the highest average abundance (Supplementary Figure 4A). Followed by *Basidiomycota* (average 2.6%) and *Mortierellomycota* (average 0.4%). Nitrogen fertilizer application reduced the abundance of *Mortierellomycota* in the rhizosphere soil of all TB varieties (*P* < 0.05). For *Ascomycota*, nitrogen application reduced its relative abundance in the rhizosphere soil of FH and YQ2, and increased the relative abundance of *Ascomycota* in the samples of XQ2 and YQ1 (*P* < 0.05). Nitrogen application decreased the relative abundance of *Basidiomycota* in FH, XQ2 and YQ1 samples, while the abundance of *Basidiomycota* in YQ2 samples increased (*P* < 0.05).

At the genus level, *Fusarium* (average 21.3%), *Penicillium* (average 15.8%), *Mycosphaerella* (average 6.7%) and *Monilinia* (average 2.9%) were the most abundant genus in all samples (Supplementary Figure 4D). Applying nitrogen fertilizer reduced the relative abundance of *Fusarium*, *Penicillium* and *Monilinia* in FH and YQ1 samples, and increased the abundance of *Mycosphaerella* (*P* < 0.05). The relative abundance of *Fusarium* in XQ2 and YQ2 under N90 was lower than that under N0, while the abundance of *Mycosphaerella* was higher under N90 than that under N0 (*P* < 0.05). For *Penicillium*, nitrogen application increased its abundance in XQ2 and decreased its abundance in YQ1 and YQ2 rhizosphere soil (*P* < 0.05). In addition, the abundance of *Monilinia* was decreased in XQ2, but that in YQ2 was increased by applying nitrogen fertilizer (*P* < 0.05).

### 3.7. Structural differentiation of microbial community of TB

**FIGURE 4:**
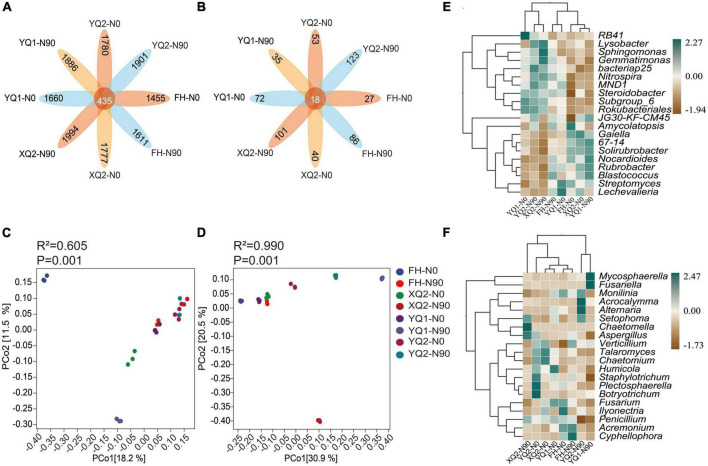
Effects of nitrogen application on the microbial community structure of rhizosphere in TB. **(A)** The OTU petal diagram of bacteria. **(B)** The OTU petal diagram of fungi. **(C)** The PCoA analysis of bacteria. **(D)** The PCoA analysis of fungi. **(E)** The heatmap of genus-level abundance clustering of bacteria. **(F)** The heatmap of genus-level abundance clustering of fungi.

In this study, unique and shared OTUs between different samples were analyzed. As seen from Figure 4A, the number of common OTUs shared by bacteria among different samples was 435, and each sample contains 1,455∼1,994 specific OTUs. At the same time, nitrogen application increased the number of specific OTUs in TB samples. The total number of common OTUs of fungi was 18, and each sample contains 27∼123 specific OTUs (Figure 4B). Nitrogen application increased the number of specific OTUs in FH, XQ2, and YQ2, but reduced that in YQ1.

Based on PCoA analysis (Figure 4C), FH and XQ2 samples under N0 and YQ1 samples under N90 were distributed on the left side of PCoA1 (*P* < 0.05), which indicating that nitrogen application had a significant impact on the distribution of FH, XQ2, and YQ1 rhizosphere bacterial communities. For fungi (Figure 4D). The samples of XQ2, YQ1, and YQ2 were distributed on the right side of PCoA1 under N90 (*P* < 0.05), which indicating that nitrogen application significantly affected the distribution of fungal communities in the rhizosphere soil of XQ2, YQ1, and YQ2.

We further illustrated the effect of nitrogen fertilization on rhizobacteria (Figure 4E). Nitrogen application increased the abundance of *Lysobacter, Gemmatimonas, bacteriap25, Nitrospira, MND1*, and *Steroidobacter* in the rhizosphere soils of FH, XQ2, and YQ2, but decreased the abundance of these bacteria in YQ1. Meanwhile, nitrogen application increased the abundance of *Gaiella*, *67-14* and *Solirubrobacter* in YQ1, but decreased the abundance of these bacteria in the rhizosphere soil of FH, XQ2, and YQ2.

As showed in Figure 4F, the application of nitrogen fertilizer could increase the abundance of fungi, *Mycosphaerella* and *Acrocalymma*, in TB rhizosphere soil. Also, nitrogen application could increase the abundance of *Talaromyces, Chaetomium, Humicola*, and *Staphylotrichum* in FH rhizosphere soil and decrease the abundance of these fungi in XQ2, YQ1 and YQ2. The abundance of *Fusarium* in TB rhizosphere soil in N90 area was lower than that in N0 area.

### 3.8. Differences in rhizosphere bacteria composition of different TB varieties

**FIGURE 5:**
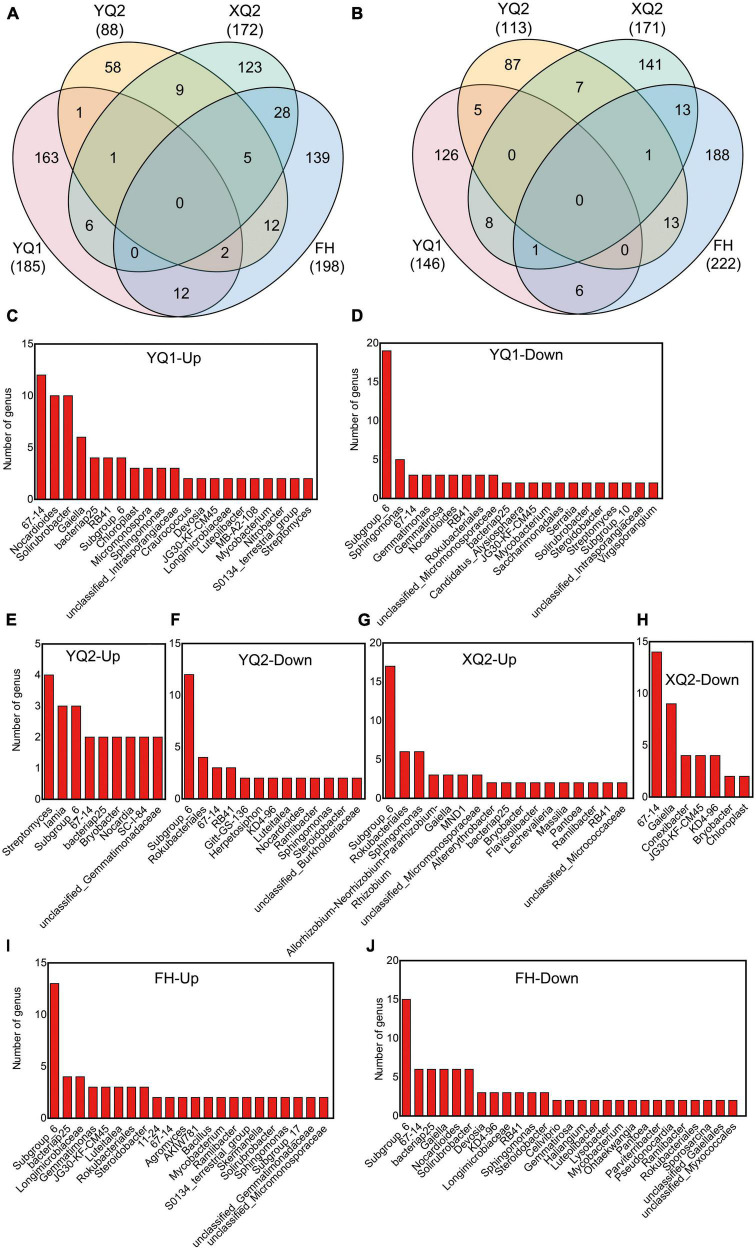
Identification of differential bacteria OTUs of TB after nitrogen application. **(A)** Venn diagram showing the enriched differential OTUs in TB. **(B)** Venn diagram showing the depleted differential OTUs in TB. **(C–J)** The specifically enriched and specifically depleted OTUs in YQ1, YQ2, XQ2, and FH.

We further compared the composition differences of rhizosphere bacteria among four TB varieties. After applying nitrogen fertilizer, it was found that 185, 88, 172, and 198 OTUs were enriched in YQ1, YQ2, XQ2, and FH, respectively, and 146, 113, 171, and 222 OTUs were depleted. 163, 58, 123, and 139 OTUs were specifically enriched in YQ1, YQ2, XQ2, and FH, respectively, and 126, 87, 141, and 188 OTUs were specifically depleted (Figures 5A, B; Supplementary Tables 1, 2). We made a taxonomic annotation on the specific enriched and depleted OTUs in TB at the genus level (Figures 5C–J). In YQ1, the number of enriched genera *67-14* was the largest (12 OTUs), followed by *Nocardioides* (10 OTUs), *Solirubrobacter* (10 OTUs), *Gaiella* (6 OTUs) and *bacteriap25* (4 OTUs), while those specifically depleted genera were *Subgroup_6* (19 OTUs), *Sphingomonas* (5 OTUs) and *67-14* (3 OTUs) (Figures 5C, D). *Streptomyces* (4 OTUs), *Iamia* (3 OTUs) and *Subgroup_6* (3 OTUs) were specifically enriched in YQ2, while *Subgroup_6* (12 OTUs), *Rokubacteriales* (4 OTUs), *67-14* (3 OTUs) and *RB41* (3 OTUs) were specifically depleted in YQ2 (Figures 5E, F). *Subgroup_6* (17 OTUs), *Rokubacteriales* (6 OTUs) and *Sphingomonas* (6 OTUs) were specifically enriched in XQ2, and *67-14* (16 OTUs), *Gaiella* (9 OTUs), *Conexibacter* (4 OTUs) and *JG30-KF-CM45* (4 OTUs) were specifically depleted (Figures 5G, H). *Subgroup_6* (13 OTUs), *bacteriap25* (4 OTUs), *Longimicrobiaceae* (4 OTUs) and *Gemmatimonas* (3 OTUs) were specifically enriched in FH, while *Subgroup_6* (15 OTUs), *67-14* (6 OTUs), *bacteriap25* (6 OTUs), *Gaiella* (6 OTUs), *Nocardioides* (6 OTUs) and *Solirubrobacter* (6 OTUs) were specifically depleted (Figures 5I, J).

### 3.9. Differences in rhizosphere fungi composition of different TB varieties

**FIGURE 6:**
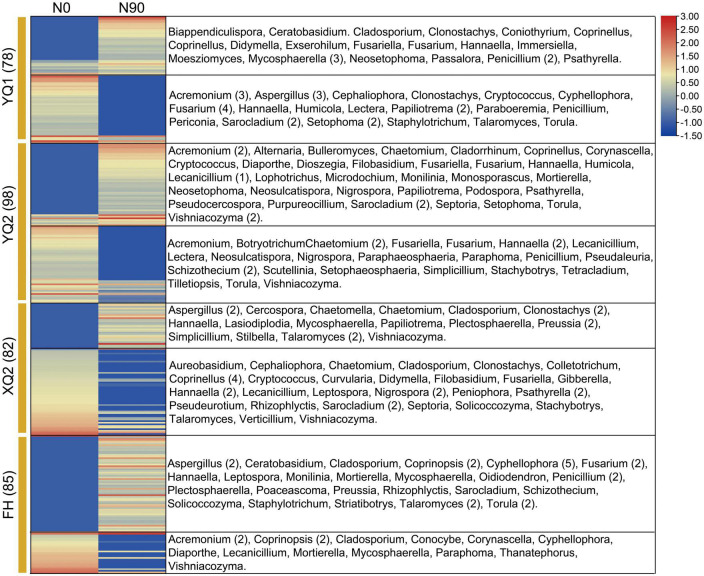
The specifically enriched and depleted OTUs in YQ1, YQ2, XQ2, and FH.

After applying nitrogen fertilizer, it was found that 60, 86, 55, and 103 OTUs were enriched in YQ1, YQ2, XQ2, and FH, respectively, and 84, 75, 82, and 49 OTUs were depleted. 35, 51, 29, and 61 OTUs were specifically enriched in YQ1, YQ2, XQ2, and FH, respectively, and 43, 47, 53, and 24 OTUs were specifically depleted (Supplementary Figures 5A, B; Supplementary Tables 3, 4). The fungal genera specifically enriched in YQ1 were *Mycosphaerella* (3 OTUs), *Penicillium* (2 OTUs), *Immersiella* and *Fusariella*; while *Fusarium* (4 OTUs), *Acremonium* (3 OTUs), *Aspergillus* (3 OTUs), *Aspergillus* (3 OTUs) and *Papiliotrema* (2 OTUs) were specifically depleted (Figure 6). Specifically enriched genus such as *Acremonium* (2 OTUs), *Sarocladium* (2 OTUs), *Vishniacozyma* (2 OTUs) and *Monosporascus* were found in YQ2, while *Chaetomium* (2 OTUs), *Schizothecium* (2 OTUs) and *Hannaella* (2 OTUs) were specifically depleted (Figure 6). *Aspergillus* (2 OTUs), *Clonostachys* (2 OTUs), *Preussia* (2 OTUs) and *Talaromyces* (2 OTUs) were specifically enriched in XQ2, while *Coprinellus* (4 OTUs), *Hannaella* (2 OTUs), *Nigrospora* (2 OTUs), *Psathyrella* (2 OTUs) and *Sarocladium* (2 OTUs) were specifically depleted (Figure 6). In addition, *Cyphellophora* (5 OTUs), *Aspergillus* (2 OTUs), *Penicillium* (2 OTUs) and *Torula* (2 OTUs) were specifically enriched in FH, while *Acremonium* (2 OTUs), *Coprinopsis* (2 OTUs), *Cladosporium* and *Conocybe* were specifically depleted (Figure 6).

### 3.10. Integrating genomic and transcriptome analysis to identify the candidate genes involved in plant–microbe interactions

**FIGURE 7:**
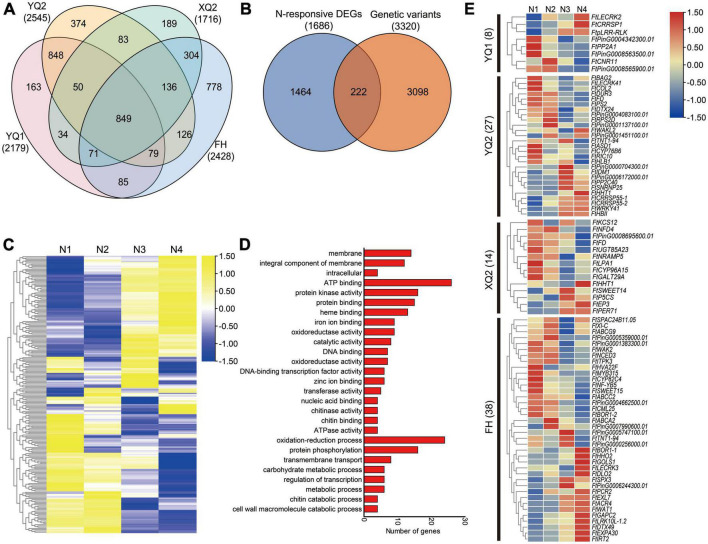
Combining genome and transcriptome screening for candidate genes involved in plant-microbe interactions. **(A)** Venn diagram showing the genes with sequence variation among different genotypes. **(B)** Venn diagram showing the nitrogen-responsive differentially expressed genes with sequence variation **(C)** Expression analysis of the 222 overlapping genes under four nitrogen conditions. N1, N2, N3, and N4 indicate the roots treated with 10, 100, 1,000 and 10,000 μM KNO_3_, respectively. **(D)** GO enrichment analysis of 222 overlapping genes. **(E)** Expression analysis of specific-varied nitrogen-responsive genes in different TB varieties.

The differences in genome level among the four TB varieties were analyzed by genomic re-sequencing. The genomes of XQ and FH were re-sequenced in our previous study, and 1,716 genes in XQ and 2,428 genes in FH showed genome sequence variation compared with Pinku 1 (reference genome), respectively (Liu et al., 2023a). In this study, the genome of YQ1 and YQ2 were analyzed by genomic re-sequencing. The SNP, InDel, CNV and SV were examined by comparing the genomes of YQ1 and YQ2 with the high-quality sequencing data of Pinku 1. A total of 484,261 SNPs, 210,348 InDels, 2,180 CNVs and 559 SVs were detected between YQ1 and Pinku 1, while 592,410 SNPs, 222,962 InDels, 2,147 CNVs and 1,146 SVs were detected between YQ2 and Pinku 1 (Supplementary Figure 6; Supplementary Table 5). The number of SNPs, InDels and SV between YQ1 and Pinku 1 was less than the number of those between YQ2 and Pinku 1. A total of 2,179 genes with genome sequence variation were found between YQ1 and Pinku 1, and 2,545 varied genes were found between YQ2 and Pinku 1 (Supplementary Table 5). Among the four varieties, in total 4,169 genes with genome sequence variation were generated, including 849 common varied genes, 163 YQ1-specific varied genes, 374 YQ2-specific varied genes, 189 XQ2-specific varied genes, 778 FH-specific varied genes and other genes (Figure 7A).

Apart from the 849 common varied genes, 3,320 varied genes’ expression in the roots under KNO_3_ treatments with different concentrations were analyzed based on the transcriptome data that was reported in our previous study (Liu C. Y. et al., 2021). A total of 222 of 3,320 varied genes showed response to nitrogen with different expression patterns (Figures 7B, C). GO enrichment analysis showed that the 222 genes were more enriched into membrane (GO: 0016020), integral component of membrane (GO: 0016021), ATP binding (GO: 0005524), protein kinase activity (GO: 0004672), protein binding (GO: 0005515), heme binding (GO: 0020037), oxidation-reduction process (GO: 0055114), protein phosphorylation (GO: 0006468) and transmembrane transport (GO: 0055085) (Figure 7D).

To analyze the different response of the four varieties’ root to nitrogen, 87 specific varied nitrogen-responsive genes were identified (Supplementary Table 6). Eight specific-varied nitrogen-responsive genes were found in YQ1, including PHLOEM PROTEIN 2-LIKE A1 (*FtPP2A1*), cysteine-rich repeat secretory protein (*FtCRRSP1*), receptor-like protein kinases (*FtpLRR-RLK* and *FtLECRK2*) and cell number regulator (*FtCNR11*) encoding genes (Figure 7E). 27 specific-varied nitrogen-responsive genes were found in YQ2, including receptor-like protein kinases (*FtWAKL2* and *FtLECRK41*), transcription factor (*FtWRKY41* and *FtCOL2*), protein phosphatase 2C (*FtPP2C40*), cysteine-rich repeat secretory protein (*FtCRRSP55-1* and *FtCRRSP55-2*) and alpha-L-arabinofuranosidase (*FtASD1*) encoding genes (Figure 7E). A total of 14 specific-varied nitrogen-responsive genes were found in XQ2, including 3-ketoacyl-CoA synthase (*FtKCS12*), beta-1, 6-galactosyltransferase (*FtGALT29A*), metal transporter Nramp5 (*FtNRAMP5*), bidirectional sugar transporter (*FtSWEET14*), delta-1-pyrroline-5-carboxylate synthase (*FtP5CS*), 7-deoxyloganetin glucosyltransferase (*FtUGT85A23*) and peroxidase (*FtPER71*) encoding genes (Figure 7E). Besides, 38 specific-varied N-responsive genes were found in FH, including transcription factors (*FtMYB315*, *FtNF-YB5* and *FtHHO2*), ABC transporter (*FtABCA2*, *FtABCG9* and *FtABCC2*), receptor-like protein kinases (*FtLRK10L-1.2*, *FtLECRK3* and *FtWAK2*), 9-*cis*-epoxycarotenoid dioxygenase (*FtNCED3*), calcium-binding protein (*FtCML25*), expansin (*FtEXPA30*), galactinol synthase (*FtGOLS1*), bidirectional sugar transporter (*FtSWEET15*) and glyceraldehyde-3-phosphate dehydrogenase (*FtGAPC2*) encoding genes (Figure 7E).

## 4. Discussion

Nitrogen was one of the key factors limiting plant growth and development and yield formation (Lin et al., 2012). The application of appropriate nitrogen fertilizer can promote the flowering and fruiting of plants, which was beneficial to the improvement of yield. Nitrogen deficiency will affect the synthesis of chlorophyll and photosynthesis in leaves, which was not conducive to the accumulation of dry matter, and eventually lead to the reduction of crop yield (Hikosaka, 2004; Wang J. et al., 2022). In this study, the agronomic characters of TB without nitrogen application were the lowest, and rational nitrogen application significantly promoted the growth and grain production of TB (Supplementary Figure 1; Figure 1), which was consistent with previous studies (Fang et al., 2018; Zhang et al., 2021). The increase of agronomic characters and yield of TB was not significant or decreased slightly under nitrogen application with > 90 kg/hm^2^ urea (Supplementary Figure 1; Figure 1). Therefore, the nitrogen fertilization with about 90 kg/hm^2^ may be reasonable for TB development and yield.

Soil microorganisms play an important role in soil nutrient cycling and decomposition of organic matter (Edwards et al., 2015; Xun et al., 2019). Rhizosphere microorganisms also play an important role in regulating plant growth (Trivedi et al., 2020). Changes in soil nitrogen content can lead to changes in microbial community composition and structure (Liu S. et al., 2021). In this study, the effect of nitrogen application on rhizosphere microbial community of TB was investigated for the first time. Compared with no nitrogen application, nitrogen application with 90 kg/hm^2^ increased the relative abundance and diversity of bacteria and fungi in the rhizosphere soil of TB. The structure of soil rhizosphere bacterial community changes with different nitrogen application rates (Gu et al., 2021). Many studies have found that the main dominant bacteria under nitrogen treatment were *Actinobacteria*, *Acidobacteria*, and *Proteobacteria* (Kavamura et al., 2018; Ren et al., 2020). *Acidobacteria* is the most abundant phylum, and its members can produce various antibiotics, which have a certain remediation effect on heavy metal pollution (Polti et al., 2014). The results of this study also showed that *Actinobacteria*, *Acidobacteria*, and *Protobacteria* were the dominant bacteria in TB rhizosphere, and the relative abundance of these bacteria was changed after nitrogen application (Supplementary Figure 3A). This study provided the first-hand information about the composition and structure of rhizosphere soil microorganisms community of TB under nitrogen application.

In this study, the relative abundance of *Nitrospira, Lysobacter*, and *Sphingomonas* in TB rhizosphere soil was increased under nitrogen application (Figure 4E). *Lysobacter* can effectively control fungal and oomycete diseases as well as cyst nematode, which can be used as a biological control agent with strong antagonistic activity (Zhao et al., 2021). *Sphingomonas* can repair environmental pollution and produce beneficial plant hormones (Li et al., 2022b). Our results also showed that nitrogen application changed the fungal community structure in the rhizosphere soil of TB, with *Fusarium* dominant in the rhizosphere soil (Supplementary Figure 4D). *Fusarium* was an important plant pathogen, and its accumulation in soil may aggravate crop diseases (Munkvold, 2017; Li et al., 2022a). It is remarkable that nitrogen application reduced the relative abundance of pathogenic fungi, *Fusarium*, in TB rhizosphere soil (Figure 4F). We also found that nitrogen application reduced the relative abundance of *Plectosphaerella* in the rhizosphere soil of TB (Figure 4F). Some studies have shown that *Plectosphaerella* may be a potential pathogenic fungus in diseased plants (Gilardi et al., 2015; Gao et al., 2021). Above analysis suggested that nitrogen fertilization with 90 kg/hm^2^ can increase the abundance of beneficial bacteria and suppress that of pathogenic fungi in TB rhizosphere soil.

Recently, investigation of the interactions between plant and rhizosphere microbe has been attracted considerable attention. Previous studies confirmed the effect of nitrogen application on rhizosphere microbial communities among different genotypes of crops is quite different (Wu et al., 2021; Qu et al., 2023). In this study, we found the composition differences of rhizosphere bacteria and fungi among four TB varieties at OTU level (Figures 5, 6). The studies in rice and maize showed that *NRT1.1B* and flavonoid biosynthetic genes mediated the plants–microbe interactions, which provide the cue for understanding of the differences on rhizosphere microbial communities among different genotypes at molecular level (Zhang J. et al., 2019; Yu et al., 2021). However, there is no report on molecular mechanism of plants–microbe interactions under nitrogen supply in other plants including TB. In this study, the candidate genes involved in plants–microbe interactions in response to nitrogen fertilizer were identified by integrating genomic and transcriptome analysis. 87 specific nitrogen-responsive genes with sequence variation were identified, including eight, 27, 14, and 38 specific-varied nitrogen-responsive genes in YQ1, YQ2, XQ2, and FH, respectively (Supplementary Table 6). Some genes encoding receptor-like kinases (RLKs), WRKY, MYB and Nramp were found (Figure 7). RLK activates plant immunity by activating plant hormones, and changes in salicylic acid signaling alter the community structure of plant rhizosphere microorganisms (Li et al., 2019, 2020; Barka et al., 2023). It is found that the *RLK* gene, *RcWAK4*, can enhance the resistance of rose to *Botrytis* resistance, and the NO signal pathway mediated by *AtWRKY27* improves the defense ability to *R. solanacearum* (Mukhtar et al., 2008; Liu X. et al., 2021; Wani et al., 2021). A *MYB* gene, *GsMYB10*, can change soybean (*Glycine max* L.) rhizosphere environment and shape the structure of rhizosphere microbial community (Liu et al., 2023c). In addition, Nramp is a kind of protein capable of transporting metal ions, which involves in the distribution of microorganisms in rice rhizosphere soil (Sasaki et al., 2012; Li et al., 2023). In general, different TB varieties have distinct specific genes that respond to nitrogen, and the expression of these genes may change the structure of microorganisms of rhizosphere soil. However, the functions of these candidate genes in the TB root–microbe interactions are needed to be investigated in the future.

## 5. Conclusion

In conclusion, the effects of nitrogen application on growth, as well as chemical properties and microbial community of rhizosphere soil were investigated in this study. Nitrogen fertilization could promote the growth of TB and significantly increase the content of available nitrogen in the rhizosphere soil. At the same time, nitrogen application with 90 kg/hm^2^ changed the distribution of microorganisms in the rhizosphere soil, and increased the relative abundance and diversity of bacteria and fungi in the rhizosphere soil of TB. Nitrogen application increased the abundance of beneficial bacteria and suppressed that of pathogenic fungi in TB rhizosphere soil. Our further study showed that there were some specific rhizosphere microorganisms of different TB varieties. Furthermore, 87 specific nitrogen-responsive genes with sequence variation, such as *FtpLRR-RLK*, *FtNRAMP5*, and *FtMYB315* genes, were identified in four varieties by integrating genome and transcriptome analysis, which may be responsible for different TB varieties to recruit specific microorganisms in the rhizosphere. This study will provide theoretical basis for further investigating the mechanism of nitrogen fertilizer in regulating the yield of TB.

## Data availability statement

The datasets presented in this study can be found in online repositories. The names of the repository/repositories and accession number(s) can be found in the article/Supplementary material.

## Author contributions

CL, HL, and QQ designed the experiments and provided financial support. QQ, DX, QL, HW, YW, QW, XY, LJ, YF, BL, YL, and HL performed data processing. CL, HL, and QQ wrote the manuscript. All authors read and approved the final manuscript.

## Footnotes

1
http://mbkbase.org/Pinku1/
