# Supplementary material for: Interkingdom multi-omics analysis reveals the effects of nitrogen application on growth and rhizosphere microbial community of Tartary buckwheat
Source: Front Microbiol. 2023 Sep 14;14:1240029. doi: 10.3389/fmicb.2023.1240029 (PMC10536138; doi:10.3389/fmicb.2023.1240029)
Supplement: Supplementary file 2 [file Data_Sheet_1.PDF]

Supplementary Material

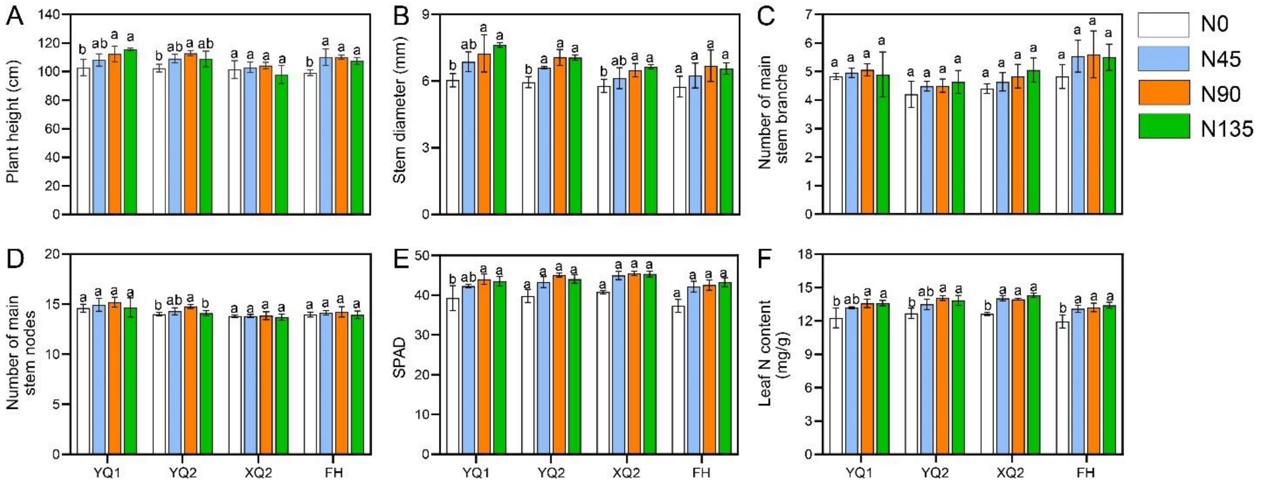

Supplementary Figure 1. Effect of nitrogen application on agronomic traits of TB. (A) The plant height. (B) The stem diameter. (C) The number of main stem branches. (D) The number of main stem nodes. (E) The SPAD. (F) The leaf nitrogen content. Different treatments marked with different lowercase letters showed significant difference ($P < 0.05$).

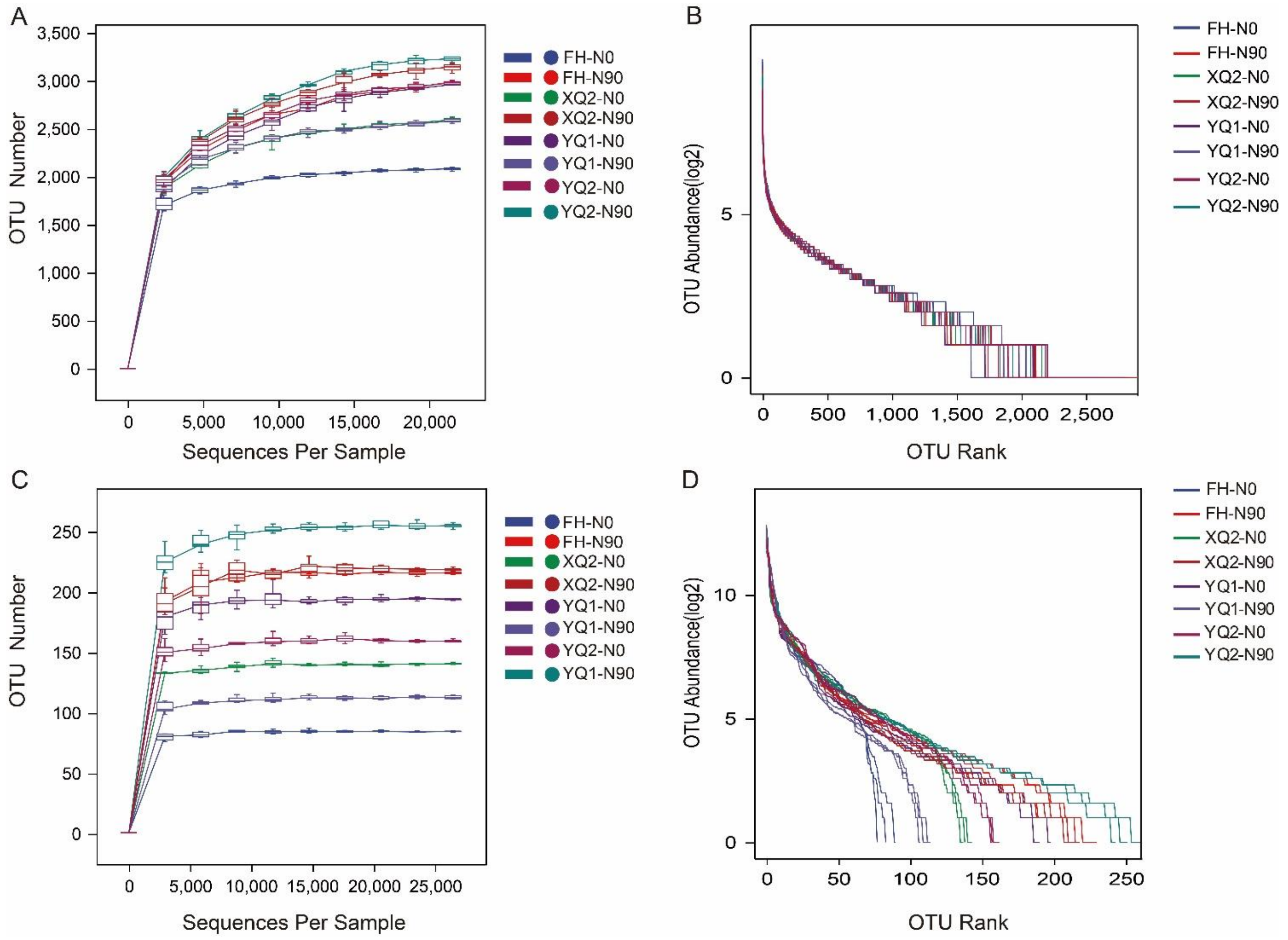

Supplementary Figure 2. The sparse curves and grade abundance curves of bacteria and fungi among different samples. (A) The sparse curve of bacteria. (B) The grade abundance curves of bacteria. (C) The sparse curve of fungi. (D) The grade abundance curves of fungi.

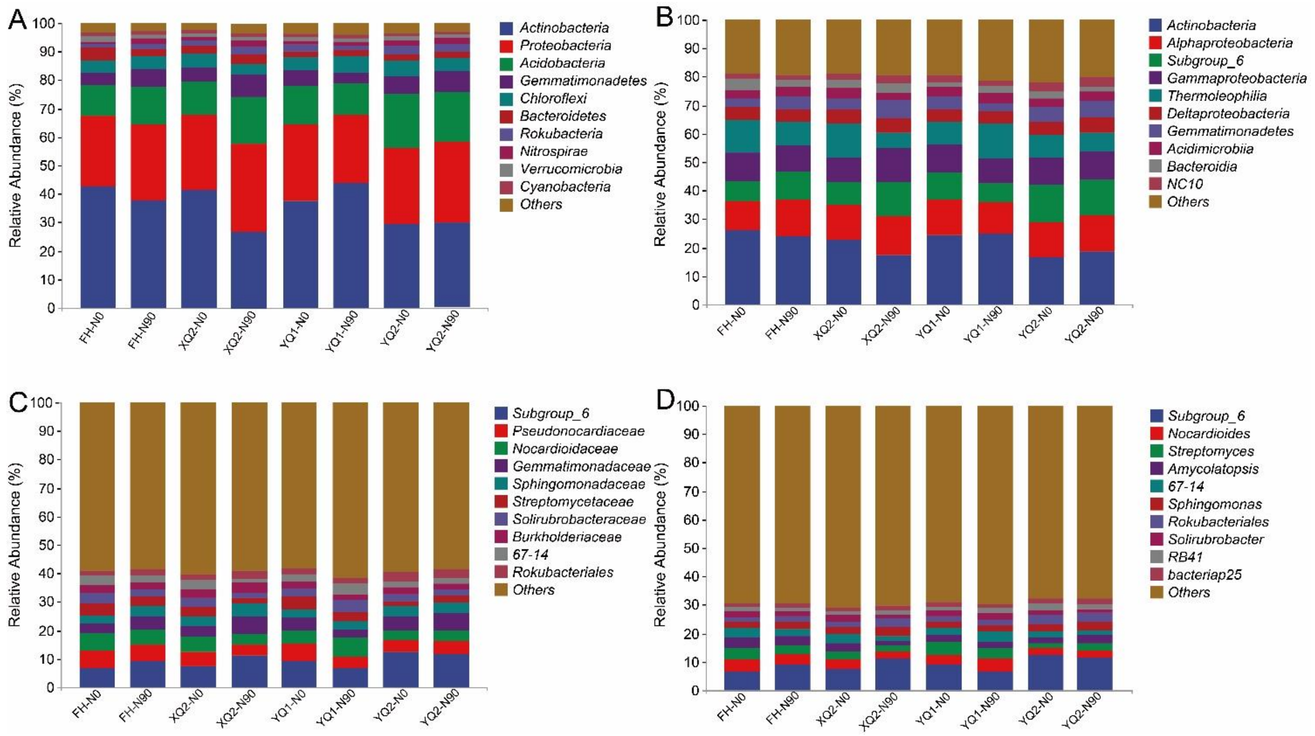

Supplementary Figure 3. The relative abundance of bacteria at the level of phylum (A), class (B), family (C) and genus (D) under different nitrogen levels.

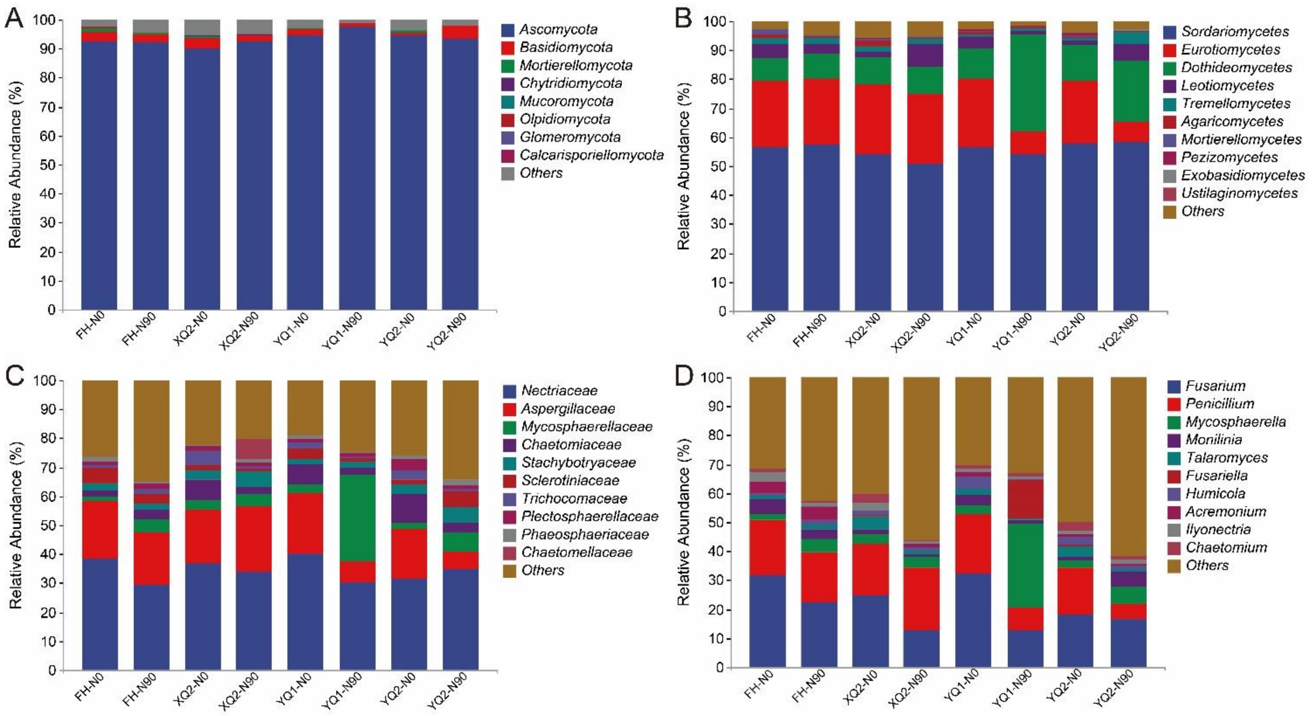

Supplementary Figure 4. The relative abundance of fungi at the level of phylum (A), class (B), family (C) and genus (D) under different nitrogen levels.

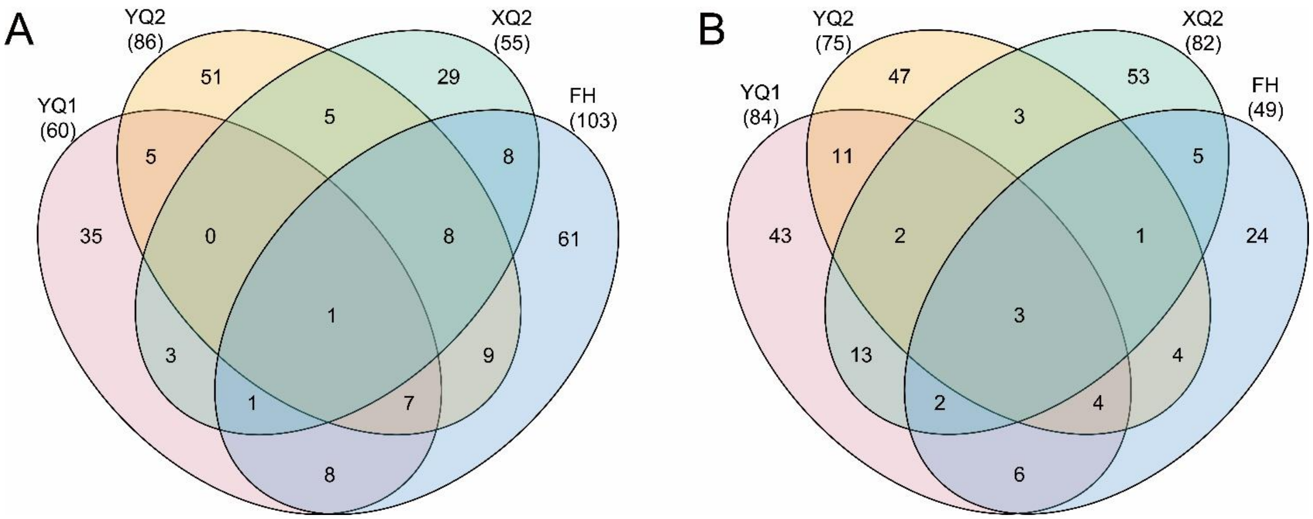

Supplementary Figure 5. Identification of differential fungi OTUs of TB after nitrogen application. (A) Venn diagram showing the enriched differential OTUs in TB. (B) Venn diagram showing the depleted differential OTUs in TB.

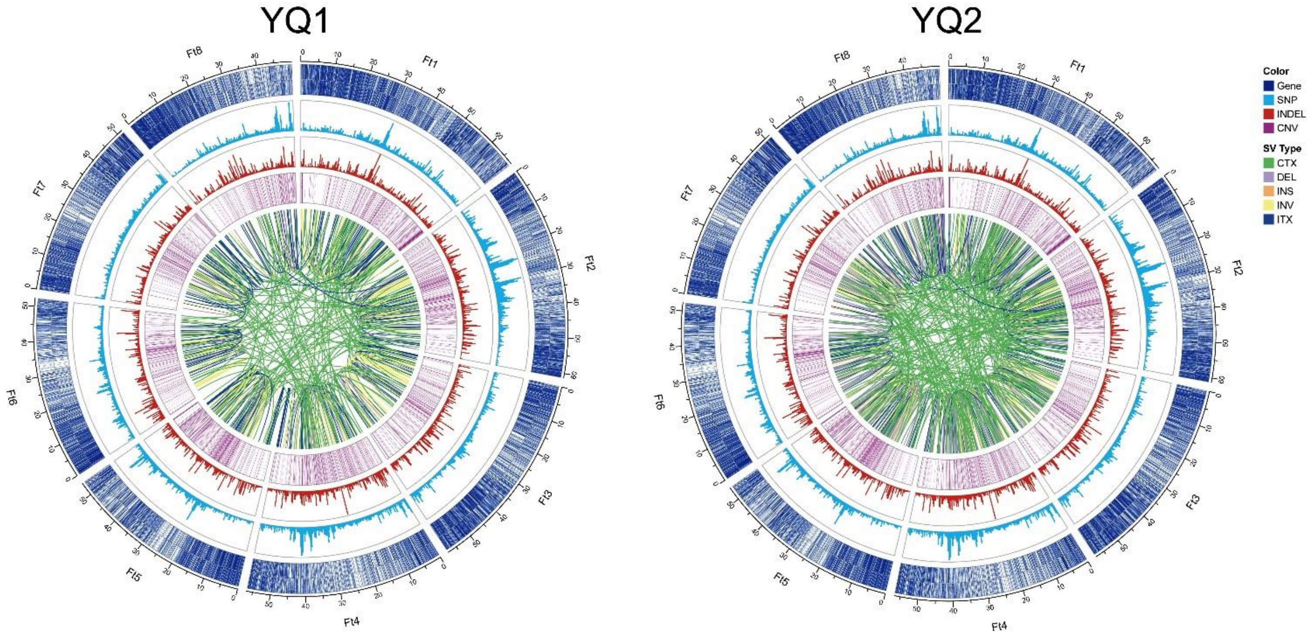

Supplementary Figure 6. The distribution of genetic variants of two TB varieties was delineated using the Circos program.
